# Cryoprecipitate and platelets-rich-plasma as a combined assisted therapy for burns: A promoted case series for future trials

**DOI:** 10.1016/j.ijscr.2023.108878

**Published:** 2023-10-02

**Authors:** Wael Barazi, Sarya Swed, Mohammad Badr Almoshantaf, Hidar Alibrahim, Haidara Bohsas

**Affiliations:** a Department of Plastic Surgery, Damascus Hospital, Damascus, Syria; b Faculty of Medicine, Aleppo University, Aleppo, Syria; c Department of Neurosurgery, Ibn-Alwalid Hospital, Homs, Syria

**Keywords:** Autografts, Burns, Cryoprecipitate, Platelet-rich plasma

## Abstract

**Introduction:**

In this paper, we present the first application of split-thickness skin autografts soaked in a combination of platelet-rich plasma (PRP) and cryoprecipitate for four cases of second and third-degree burns.

**Case presentation:**

We describe four cases of second and third-degree burns in males aged 35, 10, 24, and 5 years, respectively. The total body surface area (TBSA) affected in these cases ranged from 10 % to 35 %. The burn areas included the entire upper and lower back, the lower limbs, and the head, with involvement of the outer table of the calvarium according to Harrison's classification. To expedite wound healing, we applied split-thickness autografts soaked in a mixture of cryoprecipitate and PRP. Additionally, we covered the grafts with dressings soaked in the same mixture, resulting in successful graft acceptance and improved burn healing.

**Discussion:**

Skin wound healing involves increased angiogenesis, re-epithelialization, and modulation of inflammation. PRP has been shown to enhance re-epithelialization, a crucial process in skin wound healing. However, there is a lack of studies on the role of cryoprecipitate in re-epithelialization. Therefore, we propose the use of autologous skin grafts soaked in a combination of cryoprecipitate and PRP to expedite healing.

**Conclusion:**

This case series demonstrates that the use of split-thickness autografts soaked in a mixture of cryoprecipitate and PRP significantly improves and accelerates burn healing while contributing to acceptable graft outcomes.

## Highlights

• Many studies have shown satisfactory results with auto-grafts after administering PRP or cryoprecipitate to patients with severe burns
• Combining PRP and cryoprecipitate in a one solution is the main point in our case-series
• Careful placement of the grafts with good debridement are crucial for better graft acceptance rate.

## 1 Introduction

Burn injuries are traumatic events frequently encountered in emergency healthcare settings, presenting substantial challenges due to their potential for severe disability, high mortality rates, prolonged hospital stays, intricate rehabilitation processes, and significant financial burdens on patients and healthcare systems [1]. It is noteworthy that burn-related fatalities account for approximately 180,000 lives each year, with a staggering 90 % of these fatalities occurring in low- and middle-income countries that often lack access to well-equipped and organized burn care facilities [1].

The initial management of burn patients requires swift interventions, including airway maintenance, immediate cooling of the affected area, administration of supplemental oxygen, vigilant fluid resuscitation with continuous monitoring, and comprehensive pain control. Subsequent treatment stages encompass a range of measures, such as laboratory assessments, continuous cardiac and oxygen saturation monitoring, infection prophylaxis, appropriate antibiotic therapy, and, when necessary, minimally invasive or reconstructive surgical procedures aimed at optimizing wound healing [2]. Core principles critical to restoring both function and aesthetics in burn patients include wound coverage, meticulous site assessment, judicious technique selection, and meticulous method preparation. Achieving these objectives is possible through various approaches, including direct wound closure, grafting techniques, tissue expansion, and flap utilization [2].

In this article, we present a retrospective case series of four burn patients who received innovative treatment involving split-thickness skin autografts soaked in a solution containing a combination of platelet-rich plasma (PRP) and cryoprecipitate. These cases were conducted at Damascus Hospital, with a specific focus on evaluating the effectiveness and feasibility of this novel therapeutic approach. The application of autografts soaked in PRP and cryoprecipitate represents a distinctive and innovative intervention in burn management, one that has received limited attention in existing medical literature. We believe that this novel technique holds significant promise for enhancing burn patient care and may contribute to improved graft outcomes in the future. Furthermore, this case series adheres to the rigorous PROCESS criteria, ensuring transparent and comprehensive reporting of case series data [3].

## 2 Case Series

**Fig. 1:**
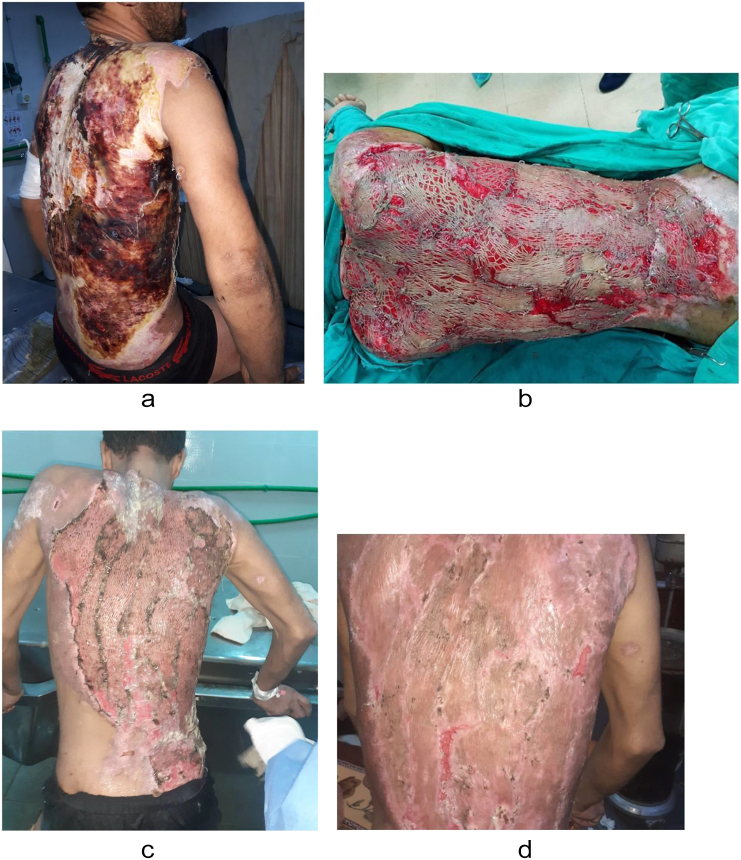
a-Case 1 before treatment; b-Case 1 in operation; c-Case 1 1st postoperative dressing; d-Case 1: 3 weeks postoperative.

Case 1 A 35-year-old male presented with accidental second and third-degree electrical burns affecting 30 % of his total body surface area (TBSA), primarily on the entire upper and lower back (Fig. 1a). In the surgical procedure (Fig. 1b), extensive debridement of the back was performed, and approximately 20 % of TBSA grafts from the lower limbs were used for autografting after graft expansion.

**Fig. 2:**
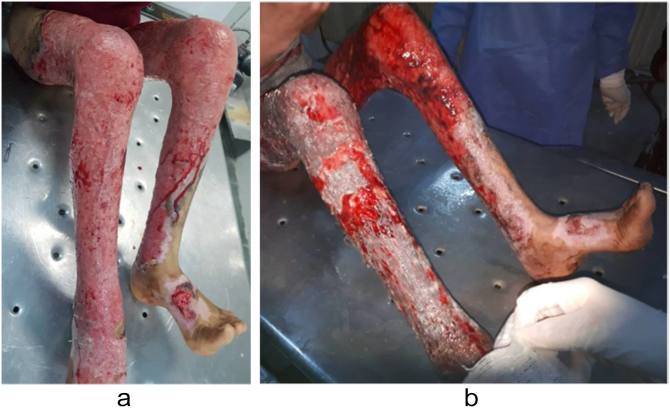
a-Case 2 before treatment; b-Case 2 1st postoperative dressing.

Case 2 A 10–year-old male child presented with accidental second and third-degree burns involving 35 % of TBSA, primarily affecting the lower right limbs (Fig. 2a).

**Fig. 3:**
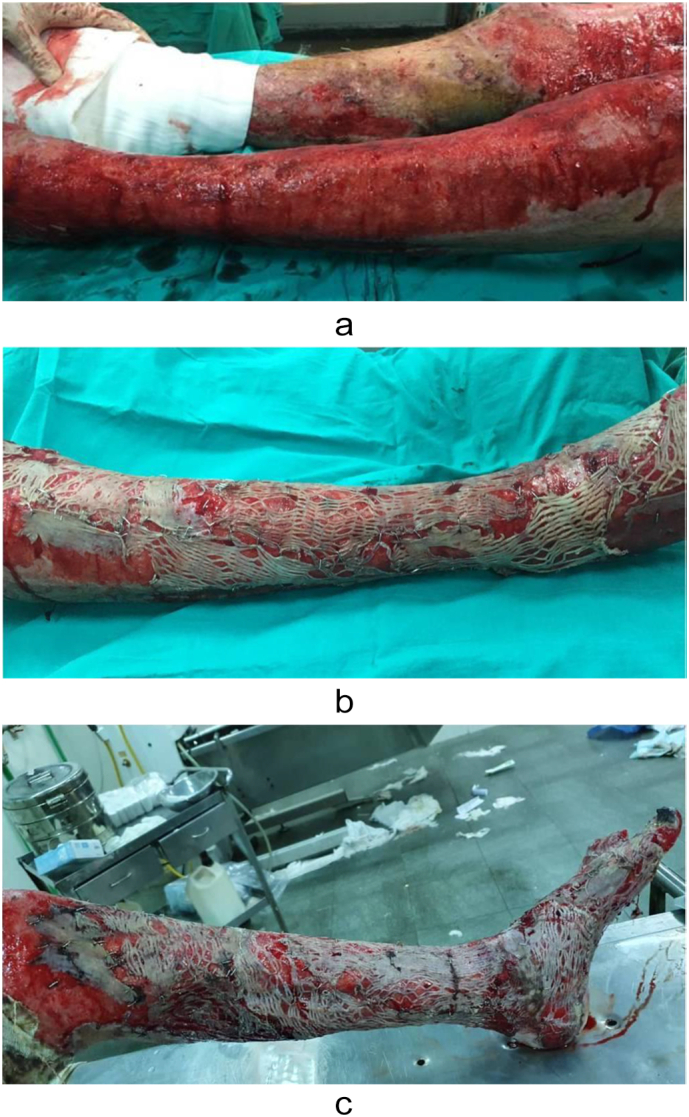
a-Case 3 before treatment; b-Case 3 in operation; c-Case 3 1st postoperative dressing.

Case 3 A 24-year-old male presented with accidental second and third-degree burns affecting 10 % of TBSA, mainly involving the lower left limb (Fig. 3a). During the surgical procedure (Fig. 3b), split-thickness grafts were used for autografting.

**Fig. 4:**
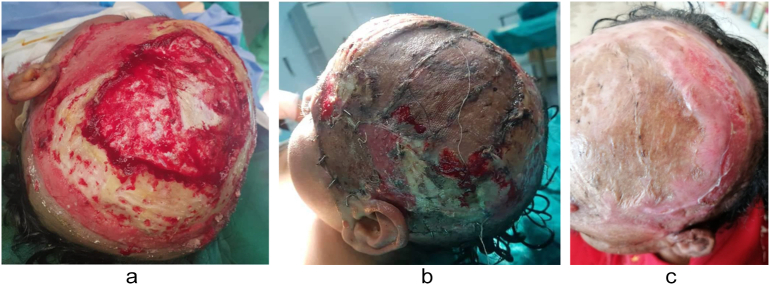
a-Case 4 before treatment; b-Case 4 in operation; c-Case 4 after 4 weeks postoperative.

Case 4 A 5–year-old male child presented with accidental third-degree burns affecting the head, with involvement of the outer table of the calvarium (Fig. 4a). An urgent operation was performed, involving surgical debridement of all devitalized scalp tissue and excision of the outer table of the calvarium, resulting in a 6 × 7 cm bone exposure. This area was covered with split-thickness autografts (Fig. 4b).

**Fig. 5:**
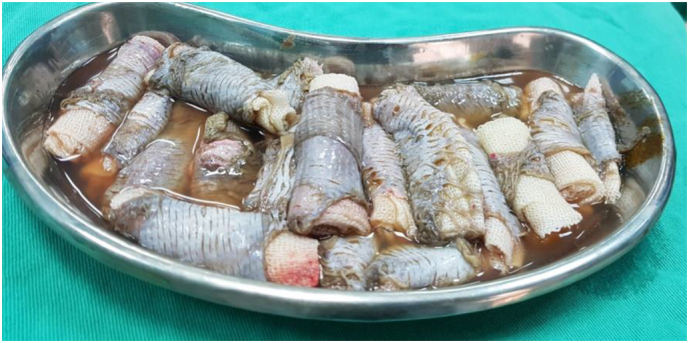
Split-thickness grafts soaked in trey of cryoprecipitate and PRP.

In all cases, the burn patients were initially referred to the plastic surgery department, where first aid and resuscitation were administered. Continuous cold-water wash was applied, and the patients were monitored using a Holter monitor. Prior to the operation, a large kidney tray was prepared, filled with a 1:1 ratio of freshly defrosted cryoprecipitate and PRP using the Imbibition method. Split-thickness skin grafts, whether meshed or not, were soaked in this solution for at least 5 min after cultivation and debridement (Fig. 5). The grafts were gently placed onto the raw skin and secured with a stapler. The entire wound area, on top of the grafts, was covered with multiple surgical gauzes soaked in the same solution. Dressings were changed between the 3rd and 5th postoperative days and again between the 10th and 15th postoperative days, after which the grafts were exposed to air. In the first three cases, the first dressing change showed a high graft success rate (Fig. 1c, 2b, 3c). *Re*-epithelialization was clearly observed on the 7th postoperative day, and patients reported a reduction in pain from severe to none, as assessed using the Numeric Rating Scale (NRS). By the 3rd postoperative week (Fig. 1d), the graft results were satisfactory, with minimal dehiscence. Additionally, for the evaluation of healing outcomes, Landry's healing index was used as a supplementary assessment tool throughout the study. On the other hand, the first dressing change for the fourth case was not recorded. However, 4 weeks postoperatively, the patient presented with a graft acceptance rate of approximately 90 %, with no other symptoms (Fig. 4c).

All patients included in this study had no significant medical history and received regular medical follow-up during their hospitalization, including ICU monitoring.

## 3 Discussion

This case series represents the first documented use of split-thickness grafts soaked in a combination of cryoprecipitate and platelet-rich plasma (PRP) for treating second and third-degree burns. Cryoprecipitate, derived from blood plasma, contains high levels of fibrinogen, von Willebrand factor, factors VIII, and XIII. Previous reports have indicated various applications of cryoprecipitate, including its use in surgical bleeding, uremic bleeding, liver diseases, hemostatic abnormalities, acquired hypofibrinogenemia, congenital fibrinogen deficiencies, and Von Willebrand disease [4].

Similarly, PRP, derived from autologous blood, is rich in growth factors and platelets. PRP contributes to wound healing and regeneration by releasing multiple growth factors and cytokines, such as basic fibroblast growth factor (bFGF), platelet-derived growth factor (PDGF), insulin-like growth factor-1 (IGF-1), vascular endothelial growth factor (VEGF), and transforming growth factor-β (TGF-β) [5]. PRP has found applications in various medical fields, including plastic surgery, cardiac surgery, orthopedics, sports medicine, gynecology, urology, and medical aesthetics. A published meta-analysis has indicated that PRP's hemostatic, adhesive, antibacterial, and healing properties can enhance skin graft success rates and reduce complete graft loss [6].

Successful skin wound healing relies on modulating inflammation, increasing angiogenesis in transplanted skin flaps, and local wound re-epithelialization. In our study, we presented four cases of second and third-degree burns, and we hypothesized that the application of split-thickness autografts soaked in combined cryoprecipitate and PRP after debridement could expedite healing. It is well-established that split-thickness skin grafts represent a suitable treatment option for severe burns [7].

However, alternative methods for wound coverage in burn patients have been explored in previous studies. For instance, cellular fish skin grafts (FSG) have been shown to improve deep partial-thickness (DPT) burn wound healing, replacing the need for autografts and allogeneic grafts. FSG promotes re-epithelialization without excessive contraction and leads to faster integration [8]. Additionally, the use of bioabsorbable dressings with antibacterial sprays during and after surgical repair and grafting for third-degree burns has demonstrated the potential to reduce infection, scar formation, and wound healing time [9].

Our case series provides valuable insights into the outcomes of our procedure, demonstrating favorable graft success rates and re-epithelialization within the first postoperative week, with minimal dehiscence observed. However, to enhance the comprehensiveness of our analysis, we have reviewed a pertinent study [10] that utilized fish skin grafts in burn treatment. The study showcased several advantages of fish skin grafts, including a faster integration into the wound bed, enhanced wound closure rates, and early increases in blood flow, which may contribute to improved outcomes. Histological analysis further supported these findings, with fish skin graft-treated wounds exhibiting superior re-epithelialization and reduced foreign material presence compared to other graft types. While our case series demonstrates promising results, we recognize the importance of conducting further comparative studies to ascertain the relative merits of various grafting techniques in the context of burn management. Such studies will undoubtedly contribute to a more comprehensive understanding of optimal approaches for achieving successful graft outcomes and ultimately improving patient care. Other study [11] involved a 7-year-old Japanese girl who underwent autologous patch skin grafts and subsequent autologous cultured epidermis (CEA) transplantation. Notably, their report indicated temporary CEA failure in regions without autologous patch skin grafts, highlighting the potential benefits of combined techniques. Additionally, our study has demonstrated favorable graft success rates, with re-epithelialization observed as early as the 7th postoperative day and minimal dehiscence. These findings contribute to the understanding of burn treatment modalities and underscore the promising outcomes achievable through our approach. In our case series, re-epithelialization was noticed in the first three cases between 5 and 7 days but the fourth case demonstrated after 4 weeks 90 % grafts acceptance rate. By the following up, there were improving, so it can be depended to management with similar cases, moreover, multicenter trials are needed to explore the actual mechanism of this technique in wound healing after burns upon combining Cryoprecipitate and Platelets-Rich-Plasma.

## 4 Limitations

The primary limitation of our case series design is the absence of a comparison group for assessing the new therapy in relation to control and other therapeutic approaches, including considerations such as application, cost, side effects, and outcomes. It is essential to acknowledge that case series studies carry a lower evidence level in evidence-based medicine due to their inherent limitations. Another noteworthy limitation is the relatively small sample size, with only four cases included in our study. Additionally, it is important to note that we did not incorporate any standardized pain scale or wound healing scale for our follow-up assessments. One of the major limitations of our study is that we did not perform our skin grafting procedure and standard in the same patients, which might provide a precise report of the findings of implantation our used methodology and the standard methodology treatment among the same patient and avoid bias in other effects such as burn degree, burn location, immunity status of the patient, and strength of the inflammatory response.

## 5 Conclusion

Several studies demonstrated sufficient success after administering PRP or cryoprecipitate to patients with severe burns, and when we combined the two, we obtained enhanced outcomes in terms of accelerating healing and re-epithelialization. Therefore, we propose that combined cryoprecipitate and PRP-soaked split-thickness autografts may promote quicker burn healing.

## Funding

None Declared.

## Ethical statement

Ethical approval for this study was provided by the Ethical Committee of Damascus Hospital, Damascus, Syria on 09/02/2023 Protocol n.00013.

## Consent

Written informed consent was obtained from the patient's (Case 4) parents/legal guardian for publication and any accompanying images. Consent was obtained from patients (Case 1, Case 2, Case 3) for publication and any accompanying images. A copy of the written consent is available for review by the Editor-in-Chief of this journal on request.

## Registration of research studies

1. Name of the registry: N/A
2. Unique identifying number or registration ID: N/A
3. Hyperlink to your specific registration (must be publicly accessible and will be checked): N/A

## CRediT authorship contribution statement

W.B: Main author

S.S, M.B.A, H.A, H.B: writing and editing the manuscript

## Guarantor

Mohammad Badr Almoshantaf: Baderalmoushantaf1995@gmail.com

## Declaration of competing interest

None Declared.
